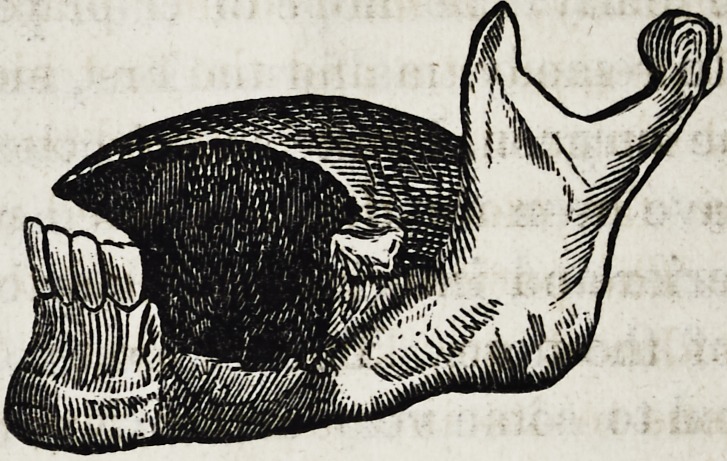# Loss of Portion of Lower Jaw and Tongue. Artificial Replacement and Restoration of Articulation

**Published:** 1868-11

**Authors:** Geo. H. Perine


### ARTICLE IX.

### *Loss of Portion of Lower Jaw and Tongue. Artificial Replacement and Restoration of Articulation*

By Geo. H. Perine, D. D. S.

The arts of medicine, surgery, and dentistry have a common origin in the demand for the alleviation of human suffering. They may justly be considered as co-ordinate branches of the art of healing. Celebrated professors, both of medicine and surgery, have written upon and practiced together all these branches. The sciences upon which these sister arts are founded are the same, ane greater or less knowledge of all essential to perfection in any. The time is past when empiricism in the dental art justified the contempt which formealy prevented affiliation. Dentistry now has her schools, her associations, her periodicals, and professors worthy the name, who would honor by their talents and skill any branch of the healing art.

The time has come when a closer union should be formed than has hitherto existed. and the higher standard of education and qualification which the leading professors of dental surgery have decided upon, is an ample guaranty and a sufficient argument for the forming of such a union.

The devotion of a department in your valuable journal exclusively to dentistry, will be hailed by the profession throughout the land as a recognition and an encouragement. It will stimulate progress not only in dentistry, but in general surgery. Many cases, that have occurred in my own practice, have shown me that these two arts can be made to mutually aid each other. Many an operation will be gladly submitted to when it becomes known that the dentist can repair the ravages of the knife, that would be refused through fear of permanent disfigurement were not the assurance felt that restoration could be made; and I have been assured by eminent surgeons that this fear is one of the serious obstacles they have to encounter in the treatment of certain diseases nvolving the maxillary bones. The art of dentistry will i also be greatly improved by the increased demand for skill and invention in this [it may be said] new field ; and everything is to be gained, nothing can be lost.

I venture to predict that you will meet with the hearty support of both dentists and surgeons throughout this country, and that the co-operation of the eminent men who belong to these professions will largely add to the value of the *Medical Gazette.*

In accordance with your request, I herewith transmit to you an account of one of a series of remarkable cases which have occurred in my practice ; and as time affords me opportunity I will prepare and forward to you reports of others.

The dental surgeon is not now, as formerly, confined to the repair of decayed teeth, the substitution of artificial ones for those already lost, or the extraction of those so far gone that there remains no hope of their preservation. He is called upon to remedy malformations, and to supplement the work of the surgeon, by the substitution of dentures for parts which have been removed, involving, oftentimes, the palate, the superior and inferior maxillary bones, the tongue and the bones of the nose. The resources of dentistry have been found equal to some very extraordinary demands, both for skill and invention. It has been my fortune to meet with a large number of cases beyond the ordinary routine of my brother practitioners, and it would perhaps be wrong for me to withhold the history of them from the profession.

In February I was called upon by Mr. —, aged forty-eight, of a bilious temperament, who several years previously had submitted to an operation for a disease of the inferior maxillary bone, which extended to and involved the left lateral portion of the tongne. The history of the case, and the precise character of the disease which necessitated the operation, were very imperfectly given by the patient, who had almost lost the power of utterance. Upon examination, I found the mouth in the following condition : A large portion of the left side of the tongue had been removed, and between the first lower bicuspid on the left and the wisdom tooth on the same side, the teeth were removed, and in their place remained a deep depression. On the opposite side of the jaw two bicuspids and one molar were gone. The surface of the tongue, where a portion had been cut away, had healed imperfectly, and there appeared to be a generally unhealthy state of the gums and the soft parts of the mouth. The breath was offensive, and the saliva was ropy. As might be expected, the general health of the patient had suffered, and he seemed anxious and worn.

My treatment was, first, to correct the morbid condition of the tissues by the use of strong astringents. As soon as the state of the parts permitted, I proceeded to take casts of the mouth. From these casts, and my previous examinations, I inferred a state of things which is approximately represented in the accompanying sketch:

The entire alveolar process between the first bicuspid and the wisdom tooth had been removed, together with a part of the body of the bone; and a large portion of the tongue had also been amputated. The action of the muscles upon the remaining portion had drawn it back, so that speech was nearly impossible, and deglutition difficult. Nature had made some feeble attempts at restoration, but so far as I could determine, the cavity left by the removal of the process had been only very partially filled by a semi-cartilaginous tissue, so that a dissection of the parts would have shown the bone nearly as it appears in the drawing.

I decided to repair this extended damage by a single denture, made of hard and soft rubber; the vulcanized rubber to sustain the artificial teeth and form a basis for the attachment of the soft rubber, with which I designed to reconstruct the tongue. The hard rubber portion filled the cavity in the jaw, and passing around and resting against the inside of the remaining alveolar process, to the right side, resting upon the gums and formed a support for the artificial teeth to be supplied on that side; the portion fitting into the cavity on the left also forming a support for the artificial teeth on that side. I moulded a piece of soft rubber into the shape of the part of the tongue which had been cut away, and extended from its borders on the right, a thin rubber membrane, forming a sack which could be slipped over and closely fitted to the remaining portion of the tongue, like a glove finger. To the posterior lower border of this portion I attached a ligament of soft rubber, and extended and attached it to the arch or plate of hard rubber above described, so that it drew equally in all directions, and covered the soft parts beneath the tongue. Finally, the hard rubber plate was attached by clasps to the dens-sapientia and the first bicuspid on the left and the second molar and first bicuspid on the right.

This denture far more than exceeded my most sanguine expectations. The patient was enabled to speak with ease, and masticate almost any kind of food. The distortion of his face previous to its introduction was remedied, and his general health much improved.—*Medical Gazette.*

## Figures and Tables

**Figure f1:**